# Supplementary figures and images for: Improved ultrastructure of marine invertebrates using non-toxic buffers
Source: PeerJ. 2016 Mar 31;4:e1860. doi: 10.7717/peerj.1860 (PMC4824901; doi:10.7717/peerj.1860)

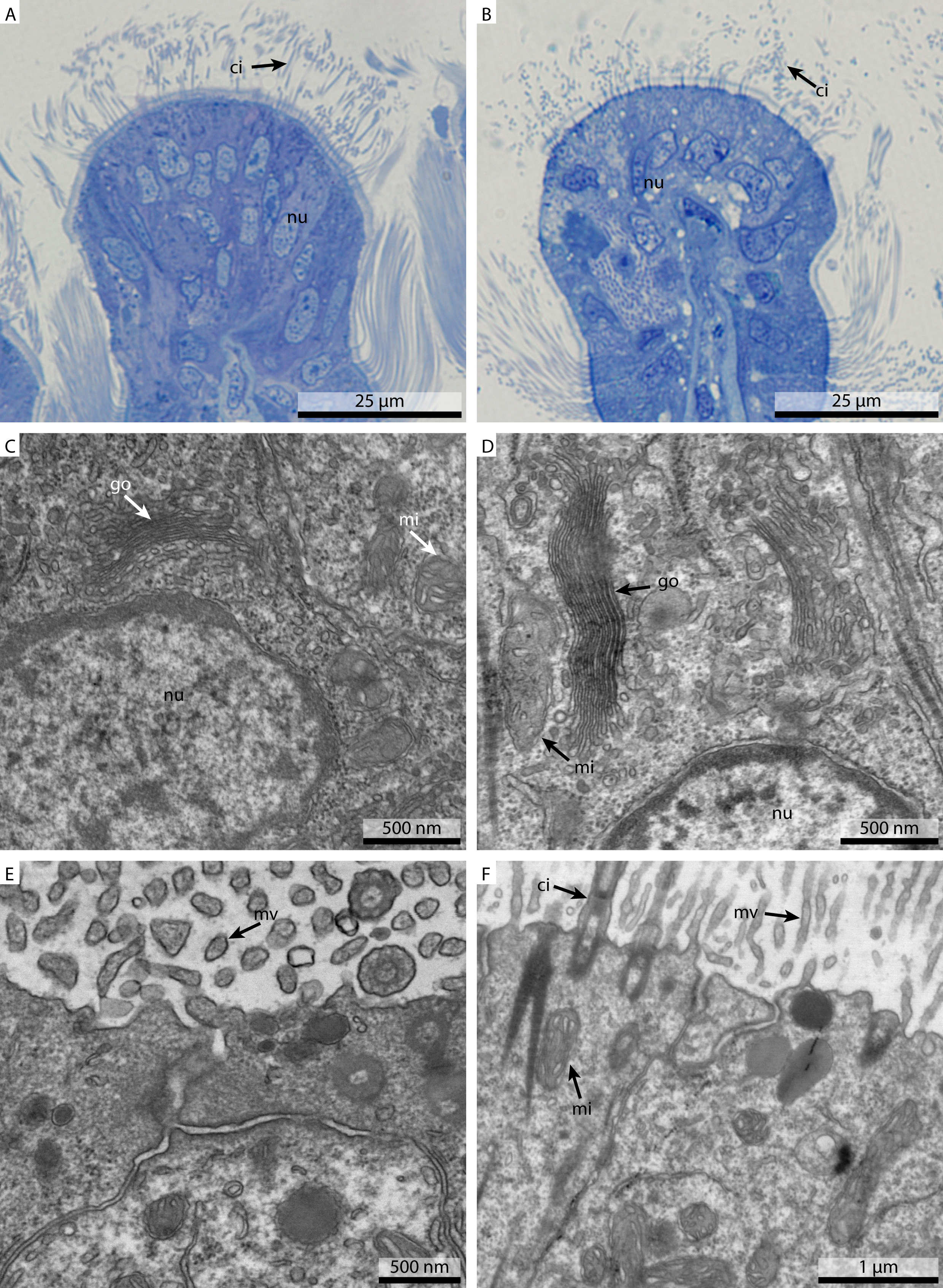

Supplement: Figure S1 — Fixative containing 3X PHEM and 2.5% GA (A, C, E) and fixative containing 1.5X PHEM, 2.5% GA and 9% sucrose (B, D, E). (A) and (B) show a light microscopic overview of the ciliated frontal surface. (C) and (D) are a close-up of the nucleus and Golgi apparatus and (E) and (F) show details of the cell surface. nu, nucleus; ci, cilia; go, Golgi apparatus; mi, mitochondria. [file peerj-04-1860-s001.png]

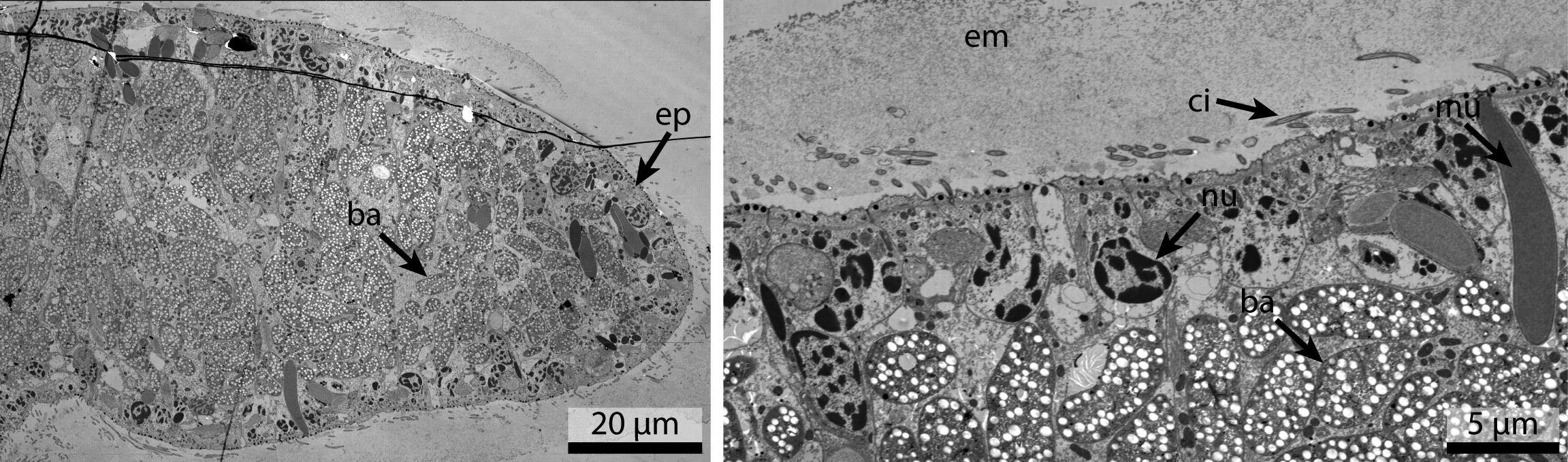

Supplement: Figure S2 — Transmission electron micrographs of Paracatenula galateia (A) is part of a cross section, showing the location of the bacteria in the bacteriocytes and (B) is a high magnification of the epidermal cell layer and the underlying bacteria. The epidermis is overlaid by a thick layer of extracellular mucus. ba, bacteria; ep, epidermis; em, extracellular mucus; nu, nucleus; ci, cilia; mu, mucus granule. [file peerj-04-1860-s002.png]

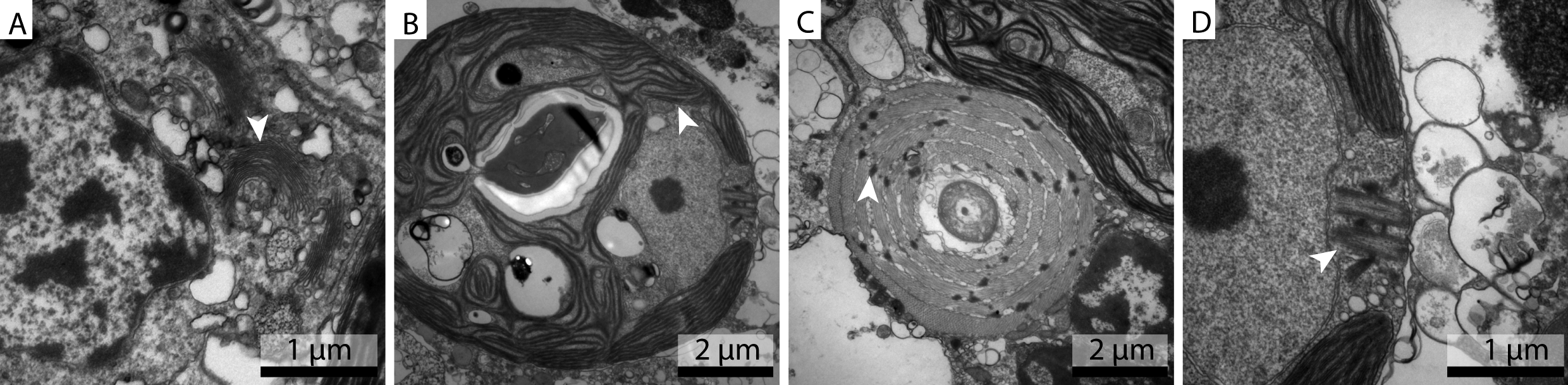

Supplement: Figure S3 — Transmission electron micrographs of Convolutriloba longifissura (A) shows part of a nucleus and multiple golgi, (B) shows the symbiotic algae, (C) shows the sagittocyst and (D) is a higher magnification of the symbiotic algae. Arrowheads indicate Golgi complexes in (A), chloroplast membrane stacks in (B), desmosomes in (C) and flagellal basal bodies in (D). [file peerj-04-1860-s003.png]

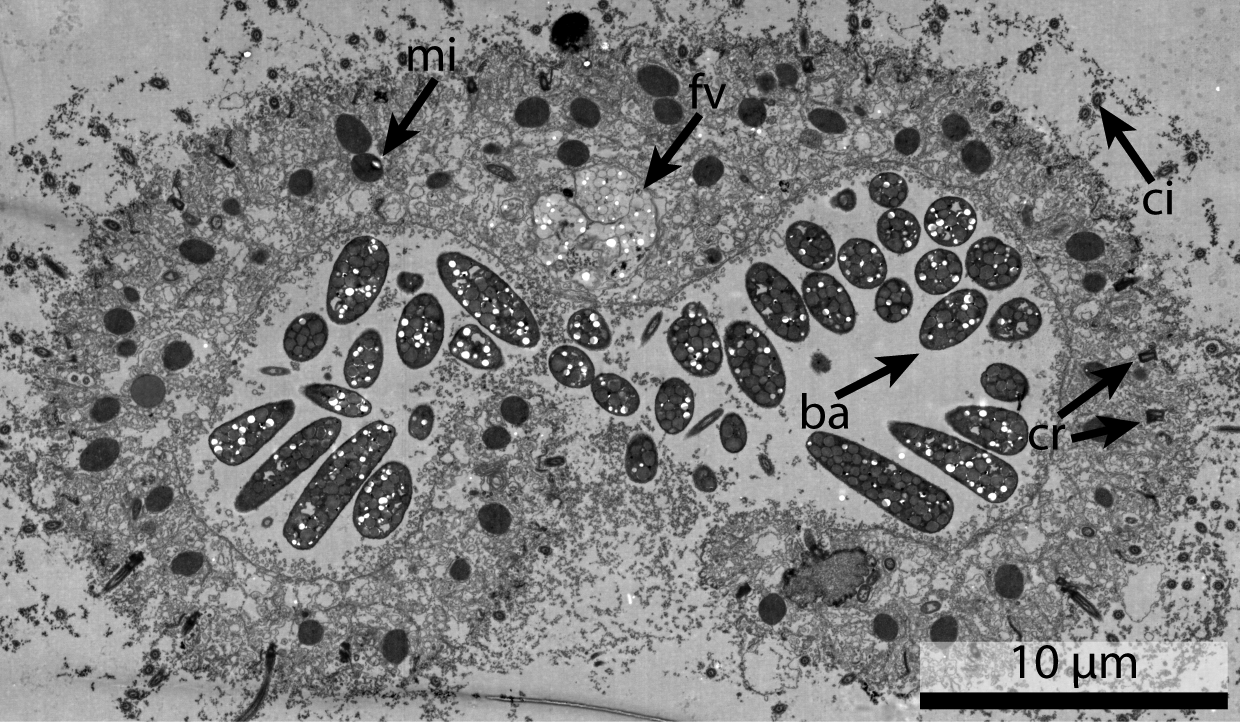

Supplement: Figure S4 — Transmission electron micrograph of Kentrophoros sp. Fig. S4 shows a cross section of the unicellular ciliate Kentrophoros, which is associated with symbiotic bacteria. ci, cilia; cr, ciliary roots; ba, bacteria; fv, food vacuole; mi, mitochondria. [file peerj-04-1860-s004.png]

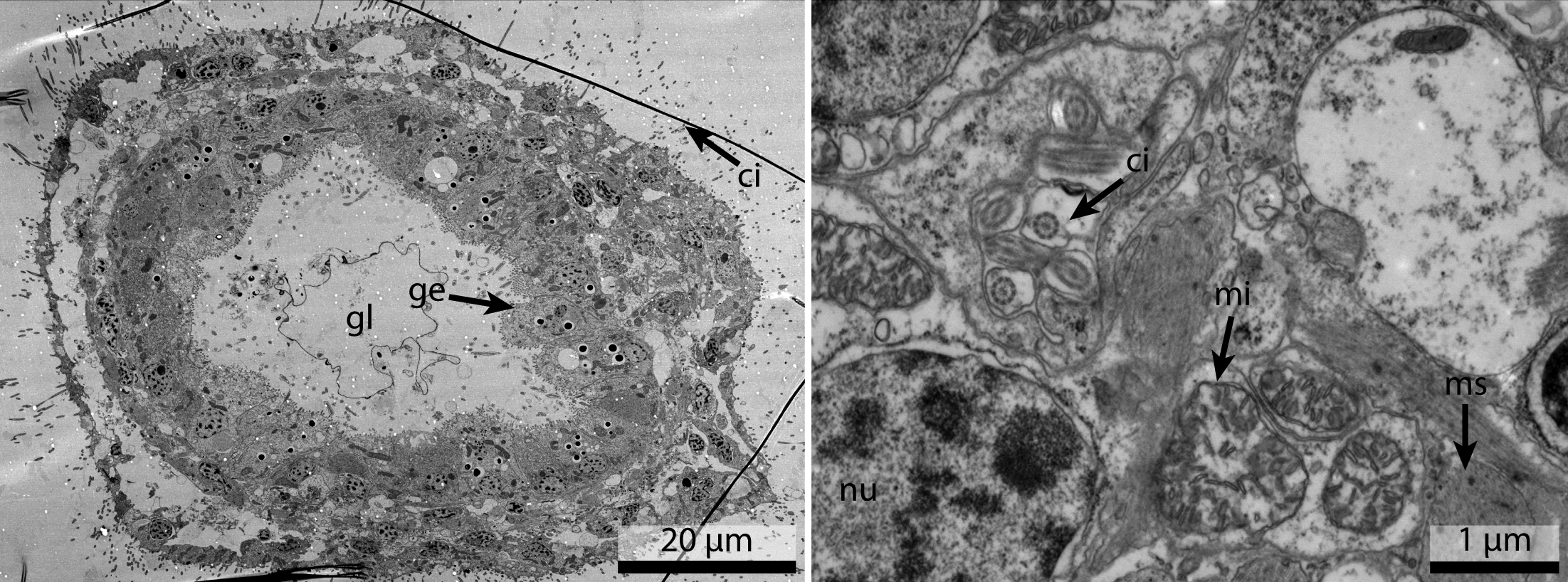

Supplement: Figure S5 — Transmission electron micrographs of Stenostomum cf. leucops (A) shows a cross section of the catenulid flatworm, with the gut lumen, and gut epithelium clearly visible. (B) is a close-up of the protonephridial duct, with multiple cilia being visible. gl, gut lumen; ge, gut epithelium; ci, cilia; nu, nucleus; mi, mitochondria; ms, muscles. [file peerj-04-1860-s005.png]
