# Supplementary material for: Improved ultrastructure of marine invertebrates using non-toxic buffers
Source: PeerJ. 2016 Mar 31;4:e1860. doi: 10.7717/peerj.1860 (PMC4824901; doi:10.7717/peerj.1860)
Supplement: Supplemental Information 1 [file peerj-04-1860-s006.docx]

# Supplementary methods:

*Convolutriloba longifissura* specimen were provided by the public aquarium Haus des Meeres – Vienna, and fixed with 2.5% Glutaraldehyde in 3X PHEM buffer for 1 hour at room temperature. After washing three times, they were post-fixed with 1% osmium tetroxide in ddH2O for one hour. The samples were then dehydrated in a graded ethanol series (50%, 70%, 100% x2) transferred into 100% dry acetone, and infiltrated with a 50/50 mixture of acetone and low viscosity resin for 30 minutes. After 2 hours of infiltration in pure LVR resin the samples were then polymerized at 60°C in the oven for 12 hours.

*Paracatenula* *galateia* and *Kentrophoros* sp. specimens were collected at 1 m depth from a depth of 1 meter at “Fisheries Beach” (16°49’25” N, 88°06’21” W) Twin Cayes, Belize. Permission for the collection and export of invertebrate animals from Belize was issued by the Ministry of Forestry, Fisheries and Sustainable Development of Belize. They were extracted from the sand by re-suspending the sediment in ample seawater and pouring the supernatant through a 63 µm-pore-size mesh sieve. The content of the sieve was transferred into a petri dish and single specimens were picked using a pipette. Samples were fixed in marPHEM for 12 hours at 4°C, washed thrice and stored in washing buffer. In the case of *Paracatenula galateia*, 0.1% Alcian Blue was added to the fixative to increase mucus retention. Post-fixation, dehydration and embedding was performed as described for *Mytilus edulis* in the main manuscript.

*Stenostomum* cf. *leucops* were picked from the cultivation dishes and fixed with 2.5% glutaraldehyde in 1X PHEM buffer overnight at 4°C. Post-fixation, dehydration and embedding was performed as described for *Mytilus edulis* in the main manuscript.

Ultrathin sections for Supplementary Figures 1, 2, 4 and 5 were imaged at 20kV on a Quanta FEG 250 scanning electron microscope (FEI Company, USA) equipped with a STEM detector using the xT microscope control software ver. 6.2.6.3123. Ultrathin sections for Supplementary Figure 3 were imaged at 80 kV on an EM 902 transmission electron microscope (Zeiss, Germany). Images were recorded with a SharpEye camera system (Olympus, Japan) using the AnalySIS 5.0 program (Olympus, Japan).
